# The influences of dried Chicory root and White lupine added to food on jejunal morphology: experimental study 

**Published:** 2018

**Authors:** Peter Makovicky, Zdenek Volek, Linda Uhlirova, Pavol Makovicky

**Affiliations:** 1 *Laboratory of Veterinary Histopathology in Komarno, Slovak Republic *; 2 *Physiology of Nutrition and Quality of Animal Product, Institute of Animal Science in Prague, Czech Republic *; 3 *Department of Biology, Faculty of Education, Selye Janos University, Komarno, Slovak Republic *

**Keywords:** Celiac disease, Gluten-free diet, Nutrition, Small bowel, Villi intestinalis

## Abstract

**Aim:**

The objective of this work was to test the effects of adding dried Chicory root and White lupine food on small bowel morphology and compare it to a standard commercial diet.

**Background:**

Various commercial gluten-free products, gluten-free raw materials and gluten-free plants are this time available on the food market, but there are still not enough information about their effect on the small bowel morphology.

**Methods:**

Altogether thirty rabbits were used in this study. The control diet (C) contained common feed components. The first experimental diet (E1) contained (per kg) 60 g of dried chicory roots instead of barley, whereas the second experimental diet (E2) was based on white lupine seeds (cv. Amiga; 120 g per kg diet) instead of the soybean meal used in the control diet. The experiment started when the rabbits were 34-days old and lasted until they were 55-days old. At the end, one jejunal small bowel tissue was sampled, and both the heights and depths of the villi and crypts were measured.

**Results:**

The highest villi were measured in the E1 (598.99 µm) group, mean in the C (590.30 µm) group and the lowest were in the E2 (563.74 µm) group. The most intense mucin villous positivity was observed in the E2 group, followed by the E1 group, and the weakest positivity was found in the visible C group.

**Conclusion:**

Chicory root has practical uses in gluten-free industries.

## Introduction

 Celiac disease (CD) is a disease that affects children and adults. It is a serious disease that is characterised by permanent intolerance to gluten. CD is currently recognised around the world, and there is substantial information available about diagnosis, prevalence, symptomatology, pathology, and pathophysiology of the disease, including other important data and problems (1, 2). At present, there is only one relevant recognized therapy, consisting in gluten-free diet. The gluten-free products are now wider in comparison with the past. In some economically advanced countries, there is a wider variety of gluten-free foods offered, but in some countries the options for individuals with CD remain narrow. Most gluten-free wheat production is based on several identical products (3). Some naturally gluten-free materials are common such as corn, rice, potato starch, pseudocereals and some less common. Additionally, a gluten-free diet involves relatively costly items, and not everyone can afford it. This is not just a problem of socially weak families but is also a concern for a wider population (4). Other naturally useful products that can be suitable for gluten-free diet are also sought. Beside, there has been a long-term effort to find some food substitutes that could be used in gluten-free food production. Part of the research is based on chemical methods of purifying traditional flour products, and some researchers have sought to find new alternatives (5-7). Based on the fact that there are some plants that are used in gluten-free food production as a food addition, we decided to test the effects of dried Chicory roots and White lupine added to food on jejunal morphology in an experimental rabbit model. White lupine is well known in gluten-free diet and it is traditional crop. On the other hand, Chicory (*Cichorium intybus, Asteraceae (Cichoriacae)*) is currently used for gluten-free diet as addition to different foods. It has an intensive bough and a yellowish white spindle root. Chicory blooms from July until September. It is often not in demand and is abundant in the lowlands (8). For healing purposes, a root is used containing fructose inulin, glycoside, choline, reducing sugars, resinous, slimy substances, and potassium salts (9). Root extracts have effects on the gallbladder, including stomach effects that are induced in the absence of gastric juice secretion (10). Although there are a whole range of results demonstrating the impact of individual gluten-free products on small bowel morphology, there is today any relevant histological information about Chicory roots effect on small bowel morphology. On the other hand, it is well know that higher doses can irritate the intestinal tract. In practice, it is not clear what doses can irritate and what have a positive effect on gastrointestinal digestion. Starting with a hypothesis of a positive effect of a dried Chicory root food additive on small bowel morphology, we compared it to the effect of a classical gluten-free White lupine food additive and a standard commercial diet in an experimental rabbit model. The objective of this work was to measure the height of villi and depth of crypts in the small bowel after feeding animals food containing dried Chicory root. 

## Methods

**Ethics**

The experiments were approved by a committee of the Institute of Animal Science (Prague-Uhrineves, Czech Republic), including the Central Commission for Animal Welfare of the Ministry of Agriculture of the Czech Republic (Prague, Czech Republic), and the experiments followed the guidelines for applied nutrition experiments in rabbits (11). 

**Experimental diets**

The three diets were formulated, and the ingredients and chemical composition of the diets are shown in Table 1. The control diet (C) contained common feed components and met the nutritive recommendations of de Blas and Mateos (12) for growing-fattening rabbits. The first experimental diet (E1) contained 60 g (per kg) of dried chicory roots instead of barley. The second experimental diet (E2) was based on white lupine seeds (Amiga; 120 g per kg diet) instead of the soybean meal. The diets were designed to have similar levels of crude protein, neutral detergent fibre (NDF), acid detergent fibre (ADF), and ether extract. The diets differed mainly with respect to the starch and fructan contents. The diets were offered as 3 mm pellets with a length of 5-10 mm. 

**Animals**

Thirty Hyplus rabbits (800 ± 113 g) were kept in the rabbit building of the Institute of Animal Science under controlled environmental conditions. Animals were weaned at 34 days of age, randomly allocated into the three groups (10 rabbits per one group). They were fed one of the three diets *ad libitum* between 34 and 55 days of age. The rabbits were individually housed in digestibility cages (50 cm x 40 cm x 42.5 cm). At the end of the experiment, all rabbits were euthanized, and one tissue sample from the small bowel was collected for histological analysis. 

**Table 1:** Composition of the diets

Ingredients (g/kg as-fed basis)	DCR	WL	Diets		
			C	E1	E2
Alfalfa meal			300	300	300
Soybean meal			70	80	0
WL			0	0	120
Wheat bran			330	330	320
Sugar beet pulp			70	50	50
Oats			150	150	120
Barley			50	0	60
DCR			0	60	0
Vitamin Supplement^1^			10	10	10
Dicalcium phosphate			5	5	5
Limestone			10	10	10
Salt			5	5	5
Determined values (g/kg as-fed basis)					
Dry matter	876	883	893	898	894
Crude protein	61	268	150	152	150
Neutral detergent fibre	83	251	406	409	404
Acid detergent fibre	82	192	215	218	224
Ether extract	35	99	48	53	57
Starch			124	78	117

Legend: DCR: Dried Chicory roots, WL: White lupine, C: Control group, E1: first experimental group, E2: second experimental group. ^1^Included per kg of feed: vitamin A, 12000 IU; vitamin D3, 2000 IU; vitamin E, 50 mg; vitamin K3, 2 mg; vitamin B1, 3 mg; vitamin B2, 7 mg; vitamin B6, 4 mg; niacinamide, 50 mg; Ca-pantothenate, 20 mg; folic acid, 1.7 mg; biotin, 0.2 mg; vitamin B12, 0.02 mg; choline chloride, 600 mg; Co, 1 mg; Cu, 20 mg; Fe, 50 mg; I, 1.2 mg; Mn, 47 mg; Zn, 50 mg; Se, 0.15 mg.

**Histological procedures**

One representative sample was subsequently taken from each animal from the jejunum, and the samples were put in a 4% formalin solution. All the samples were collected within 30 min after euthanasia and they were fixed for one week. The samples were processed according to standard histological methods. Serial slices with a 5-μm thickness were cut from each sample, and they were put on standard slides (Bamed s.r.o., Czech Republic). The first slices were stained with haematoxylin and eosin (Diapath, Italy), and the second serial set of slices were stained with Alcian blue (Diapath, Italy) for mucopolysaccharide and mucin visualisation. For the mucin positivity evaluation, a simple scoring system was used that is described in detail in Table 2. The positivity was evaluated from negative to strongly positive (0–3), and all of the measured numbers were counted per individual group and per villous. Eventually, the crypt measurements were averaged at the end. The prepared samples were evaluated under an Olympus BX50 light microscope. Villus height and crypt depth were measured using NISElements version 3.0 software (Nikon Corporation Instruments Company). The height of each villus was measured from the top of the villus to the crypt transition, and the crypt depth was defined as the invagination between two villi. The heights of 100 villi and the depths of 100 crypts were measured per rabbit. 

**Table 2:** Evaluation system for the mucin staining

Signature	Intensity of the positive staining
0	Negative.
1	Weakly positive.
2	Moderately positive.
3	Strongly positive.

**Analytical methods**

A chemical analysis of the diets, dried Chicory roots and white lupine were performed using the procedures of Van Soest *et al.* (13) for neutral-detergent fibre (NDF), acid-detergent fibre (ADF) and acid detergent lignins (ADL). The AOAC (14) International procedures were used to determine the crude protein (954.01) and starch (920.40) contents. Ether extract was determined according to AOAC (15) procedure 920.39. The dry matter content was determined in duplicate samples by drying thethem at 105 °C to a constant weight. 

**Statistical analysis**

One-way ANOVA and a Scheffé test were used to test measured values. 

## Results

**The weight of the rabbits**

The heaviest rabbits were in the E1 group (1957 g), and the lightest were in the E2 group (1846 g). The results are supported by daily feed consumption with higher feed intake in the E1 group (141.3 g/day) and approximately the same feed intake in the C (129.5 g/day) and E2 (129 g/day) groups. The weight differences are not statistically significant. 

**The heights of the villi**

The villi were influenced by diet, and there were some differences between groups at the end of the experiment. The highest villi were measured in the E1 (598.99 µm) group (*P< 0.001*) and the lowest in the E2 (563.74 µm) group. In the C (590.30 µm) group, mean values were measured (*P< 0.001*). 

**The depths of the crypts**

The results showed that the crypts were also influenced. The deepest crypts were measured in the C group (99.99 µm), (*P< 0.001*) with some negligible differences compared to the E1 (95.94 µm) (*P< 0.001*) and E2 (91.36 µm) groups. 

**The intensity of the mucin staining**

The most intense mucin villous positivity was seen in the E2 (2.44) group, following the E1 (2.18) group, and finally, the weakest positivity was visible in the C (2) group. Similar results were measured in mucin crypt positivity as well, and the most intense positivity was observed in the E1 (0.59) group with similar values in the C (0.56) and E2 (0.56) group. All the results are in shown Tables 3, 4 and 5. 

**Table 3:** Average weight of the rabbits and average daily feed consumption

Groups	W1 (g)	W2 (g)	DFC (g/day)
C	789	1903	129.5
E1	800	1957	141.3
E2	809	1846	129.0

Legend: W1: Average weight of the rabbits at day 34 of the experiment. W2: Average weight of the rabbits at day 55 of the experiment. DFC: Average daily feed consumption, C: Control group, E1: first experimental group, E2: second experimental group

**Table 4.** The heights of villi and depths of crypts

Groups	WV (µm)	DC (µm)
C	590.30^a^	99.99^a^
E1	598.99^a^	95.54^b^
E2	563.74^b^	91.36^c^
RMSE	207.28	31.99
*P*	0.001	< 0.001

Legend: WH: Weight of villi, DC: Depth pf crypts, C: Control group, E1: first experimental group, E2: second experimental group.

**Table 5:** The intensity of mucin positivity in the villi and in the crypts

Groups	VP	CP
C	2	0.56
E1	2.18	0.59
E2	2.44	0.56

Legend: VP: Villous part, CP: Crypts part, C: Control group, E1: first experimental group, E2: second experimental group.

## Discussion

Currently, it is assumed that the CD in the European population remains underdiagnosed. The truth is that it is more frequent, and compared to the past, it more often recognised in adults. As stated in the introduction, a gluten-free diet is the only truly effective therapy for CD (16). In some situations, it is also recommended to people with some other diseases, digestive difficulties, eventually non-celiac gluten sensitivity or autism spectrum disorders (17). This is controversial problem and there are several views to the gluten-free diet tasks in non-celiac autoimmune diseases. According to the Lerner *et al.* (18) this time we are far away from unravelling the mechanisms by which gluten-free diet can alleviate non-celiac autoimmune diseases initiation or progression and there are more questions than answers on this very challenging topic. It is also well known that the diet is expensive and increases food spending for people with CD. Additionally, the availability of commercial gluten-free foods and gluten-free products or raw products varies from country to country. On the other hand, there are big gaps in the gluten-free diet today (19, 20). Some manufacturers concentrate on the chemical processing of materials, such as gluten removal (21, 22). These expensive methods are often reflected in the final product price. Although some naturally gluten-free plant products are used today, the number of options remains inadequate. Chicory root fibre is a natural, prebiotic fibre and it is used in the development of gluten-free food products, including bread. Chicory root fibre can also offer significant product quality benefits in this technically challenging endeavour (23, 24). Our results show positive effects of Chicory roots on the jejunum with intense mucin production. The mucin villous intensity was higher compared to the mucin villous intensity of standard commercial diet but lower compared to the mucin villous intensity of white lupine food addition. In terms to the crypt positivity, the mucin intensity reaches the same level after Chicory roots, eventually White lupine food addition and higher comparing to the mucin depth intensity of standard commercial diet. These are very important findings, documenting the direct effects of Chicory roots and White lupine on cellular activity with mucin secretion. Mucins are glycoproteins that are common on the surfaces of intestinal mucosa. They have a protective role for epithelial tissues. So mucin production can also have preventive effects on various intestinal diseases. On the other hand, it was proved that some of them have been identified as markers of colorectal patient’s adverse prognosis (25). Two mains families can be distinguished and they are secreted mucins and membrane‐bound mucins (26). In our study we did not identify individual mucins. We assume that if the secretion of healthy cells has increased, so mucin secretion may be a positive sign of differentiation of the small bowel mucosa cells in case of malabsorption. Castellini *et al.* (27) also demonstrated that the addition of chicory in the post-weaning phase slightly affected rearing performance. The rabbits fed chicory showed a higher feed intake and daily gain. These results document the effects of chicory root on the gastrointestinal tract with progressive secretion activity. This outcome shows that chicory root could be also tested for the pharmaceutical industry and eventually widely used in the gluten-free industry. These might include further studies in their animal model that focus on the microvillus surface membrane glycol-conjugates and goblet cell mucins. Here it comes into consideration especially in early stages of small bowel regeneration and for accidental ingestion of gluten in people with CD. Additionally, it could be useful after long-term antibiotic therapy or for individuals under cytostatic treatment. Today it is known that these stages are characterised by alterations in the gastrointestinal tract with small bowel villous atrophy, and these changes are accompanied by a transient lack of food appetite. In this process, proliferation and maturation of gastrointestinal tract cells are particularly important. The Enterocytes, Goblet cells, Paneth cells, stem cells, and junctional complex have a special status in this process, including alterations in the microbiota with an ability to restore gastrointestinal tract function (28, 29). On the other hand, longer use or even concentrated chicory root use will finally irritate stomach epithelial cells, including small bowel epithelial cells and will cause gastrointestinal problems (30). Therefore, it should not be used directly, or alone, but only as an addition of a gluten-free food. It would also be useful in the gluten-free industry as a gluten-free food addition in minimal quantities. 

The Chicory root food addition documents positive effects to jejunal morphology with highest villi, including most intense mucin villous positivity comparing White lupine group and control group. 
